# Parvalbumin and neuropeptide Y expressing hippocampal GABA-ergic inhibitory interneuron numbers decline in a model of Gulf War illness

**DOI:** 10.3389/fncel.2014.00447

**Published:** 2015-01-08

**Authors:** Tarick Megahed, Bharathi Hattiangady, Bing Shuai, Ashok K. Shetty

**Affiliations:** ^1^Research Service, Olin E. Teague Veterans’ Medical Center, Central Texas Veterans Health Care SystemTemple, TX, USA; ^2^Institute for Regenerative Medicine, Texas A&M Health Science Center College of Medicine at Scott & WhiteTemple, TX, USA; ^3^Department of Molecular and Cellular Medicine, Texas A&M Health Science Center College of MedicineCollege Station, TX, USA

**Keywords:** Gulf War illness, hippocampal GABA-ergic interneurons, parvalbumin interneurons, neuropeptide Y interneurons, somatostatin interneurons, hippocampal neurogenesis, hippocampus hyperexcitability, cognitive impairments

## Abstract

Cognitive dysfunction is amongst the most conspicuous symptoms in Gulf War illness (GWI). Combined exposure to the nerve gas antidote pyridostigmine bromide (PB), pesticides and stress during the Persian Gulf War-1 (PGW-1) are presumed to be among the major causes of GWI. Indeed, our recent studies in rat models have shown that exposure to GWI-related (GWIR) chemicals and mild stress for 4 weeks engenders cognitive impairments accompanied with several detrimental changes in the hippocampus. In this study, we tested whether reduced numbers of hippocampal gamma-amino butyric acid (GABA)-ergic interneurons are among the pathological changes induced by GWIR-chemicals and stress. Animals were exposed to low doses of GWIR-chemicals and mild stress for 4 weeks. Three months after this exposure, subpopulations of GABA-ergic interneurons expressing the calcium binding protein parvalbumin (PV), the neuropeptide Y (NPY) and somatostatin (SS) in the hippocampus were stereologically quantified. Animals exposed to GWIR-chemicals and stress for 4 weeks displayed reduced numbers of PV-expressing GABA-ergic interneurons in the dentate gyrus and NPY-expressing interneurons in the CA1 and CA3 subfields. However, no changes in SS+ interneuron population were observed in the hippocampus. Furthermore, GABA-ergic interneuron deficiency in these animals was associated with greatly diminished hippocampus neurogenesis. Because PV+ and NPY+ interneurons play roles in maintaining normal cognitive function and neurogenesis, and controlling the activity of excitatory neurons in the hippocampus, reduced numbers of these interneurons may be one of the major causes of cognitive dysfunction and reduced neurogenesis observed in GWI. Hence, strategies that improve inhibitory neurotransmission in the hippocampus may prove beneficial for reversing cognitive dysfunction in GWI.

## Introduction

Almost 25–30% of 700,000 troops deployed to the Persian Gulf War-1 (PGW-1) are diagnosed with Gulf War illness (GWI), which is a chronic multi-symptom illness affecting multiple systems including the central nervous system (Binns et al., 2008, 2014; Golomb, 2008). Cognitive and mood impairments are among the conspicuous brain-related symptoms in GWI veterans and in animal models of GWI (Haley et al., 2000a,b; Steele, 2000; Odegard et al., 2013; Parihar et al., 2013; Rayhan et al., 2013; Hattiangady et al., 2014). Although several potential causes have been proposed for GWI, epidemiological and animal model investigations suggest that GWI in a significant fraction of veterans is linked to a combination of chemical exposures encountered by service personnel during the PGW-1 (Binns et al., 2008, 2014). The chemicals included pyridostigmine bromide (PB), N, N-diethyl-m-toluamide (DEET) and permethrin (PM). While PB pills were consumed daily for variable periods of time as a prophylactic agent against possible nerve gas agent attacks during the war, exposures to DEET (a mosquito repellant) and PM (an insecticide) have occurred because of their extensive use on the skin and/or uniforms with the intension of offsetting infectious diseases transmitted by insects and ticks in the desert region (Haley and Kurt, 1997; Binns et al., 2008, 2014; Institute of Medicine Gulf War and Health, 2010; Steele et al., 2012).

Concurrent exposures to even moderate doses of the above GWI-related (GWIR) chemicals for extended periods are considered perilous for CNS function because of several reasons. The drug PB is a reversible acetylcholinesterase (AChE) inhibitor and does not cross the blood brain barrier (BBB) under normal circumstances. However, in situations when BBB gets leaky (such as after exposure to stress or pesticides), PB can enter the brain (Friedman et al., 1996; Abdel-Rahman et al., 2002), which may enhance the concentration of acetylcholine (ACh) in synaptic clefts for extended periods and cause hyperexcitation of neurons. On the other hand, exposure to DEET may lead to human poisoning (Chaney et al., 2000) and PM exposure can cause sustained opening of voltage-gated sodium channels in neurons leading to repetitive discharges after a single stimulus (Narahashi, 1985). Furthermore, studies have also shown that exposure to PM can alter the expression of multiple genes in the brain including those involved with the onset of brain aging processes (Harrill et al., 2008; Carloni et al., 2013) and stress can induce epigenetic changes in the brain (Rinaldi et al., 2010; Stankiewicz et al., 2013). These issues as well as animal model and epidemiological studies have prompted the hypothesis that extensive exposure to multiple chemicals such as PB and pesticides caused GWI in most veterans (Abdel-Rahman et al., 2002, 2004; Binns et al., 2008, 2014; Abdullah et al., 2011, 2012; Torres-Altoro et al., 2011; Steele et al., 2012). Consistent with this hypothesis, our recent study in a rat model showed that concurrent exposure to GWIR-chemicals PB, DEET and PM for 4 weeks causes memory and mood impairments (Parihar et al., 2013). Furthermore, addition of mild stress during the exposure to GWIR-chemicals exacerbated the extent of impairments even though mild stress alone improved cognitive and mood function in normal animals (Parihar et al., 2011, 2013; Hattiangady et al., 2014). Additionally, memory and mood impairments were associated with significant detrimental changes in the hippocampus. The alterations comprised: (i) considerable decline in neurogenesis, a process of adding new neurons to the hippocampal circuitry that is believed to be important for making new memories and maintaining normal mood function (Deng et al., 2010; Eisch and Petrik, 2012; Cameron and Glover, 2014); (ii) chronic low-level inflammation typified by hypertrophy of astrocytes and activation of microglia; (iii) increased expression of genes that respond to oxidative stress; and (iv) moderate loss of hippocampal principal neurons in certain layers (Parihar et al., 2013; Shetty et al., 2014).

The above changes likely contribute to cognitive dysfunction seen in GWI. However, additional alterations in the hippocampus may also be involved. For example, significant loss of gamma-amino butyric acid (GABA)-ergic interneurons can promote hippocampus hyperexcitability as well as worsen cognitive function via decreased inhibitory synaptic transmission (Koh et al., 2010, 2013). To ascertain whether interneuron deficiency is among the detrimental hippocampal changes in GWI, we first exposed animals to GWIR-chemicals and mild stress for 4 weeks. Three months later, we stereologically quantified subpopulations of GABA-ergic interneurons expressing the calcium binding protein parvalbumin (PV) and neuropeptides, the neuropeptide Y (NPY) and somatostatin (SS) in the hippocampus. We focused on these three types of hippocampal interneurons because previous studies have shown that, in addition to regulating the excitability of hippocampal principal neurons; these interneuron subpopulations play roles in maintaining normal levels of neurogenesis and cognitive function (Korotkova et al., 2010; Murray et al., 2011; Borbély et al., 2013; Zaben and Gray, 2013; Song et al., 2014). Furthermore, to determine whether interneuron deficiency is allied with reduced hippocampus neurogenesis, we also evaluated newly born neurons in these rats via quantification of doublecortin-positive (DCX+) newly born neurons in the subgranular zone-granule cell layer (SGZ-GCL) of the dentate gyrus (DG).

## Materials and methods

### Animals

Young adult (~3-months old) male Sprague-Dawley rats purchased from Harlan (Indianapolis, IN, USA) were used in this study. After 2 weeks of acclimatization to the vivarium, animals were randomly assigned to either the naïve control group or the GWI group (*n* = 8 per group). Animals in GWI group received daily exposure to GWIR-chemicals PB, DEET and PM and mild stress (5 min of restraint stress) for 4 weeks. From here onwards, these rats will be referred to as “GWI-rats”.

### Application of chemicals and restraint stress

The chemical PB (1.3 mg/Kg/animal) was dissolved in 500 μl of sterile water and administered via oral gavage. The chemicals DEET and PM were administered dermally. For these, solutions of DEET (200 μl containing 40 mg/kg in 70% alcohol; Chem. Service Inc., West Chester, PA, USA) and PM (200 μl, 0.13 mg/kg in 70% alcohol; Chem. Service Inc., West Chester, PA, USA) were prepared, the fur on the back of the neck and the upper thoracic region were shaved off and the exposed skin was gently wiped with 70% alcohol. Following this, using a short plastic pipette, DEET and PM (200 μl each) were successively spread over the shaved skin. A rat restrainer (Stoelting Research Instruments, Wood Dale, IL, USA) was used for the induction of 5 min of restraint stress, as described in our previous study (Parihar et al., 2011). The doses of PB, DEET, and PM and stress were chosen based on previous studies of GWI using rat models (Abdel-Rahman et al., 2002, 2004; Parihar et al., 2013).

### Animal perfusions and tissue processing

Three months after the completion of 4 weeks of exposure to GWIR-chemicals PB, DEET and PM and 5 min of restraint stress, GWI-rats and age-matched naive control rats were subjected to terminal anesthesia with isoflurane and trans-cardiac perfusion with 4% paraformaldehyde solution in phosphate buffer (PB). Following this, the brain was carefully removed from the skull of each animal and post-fixed in 4% paraformaldehyde for ~16 h at 4°C. The brain tissues were next treated with 30% sucrose solution in PB until it sank to the bottom, and thirty-micrometer thick cryostat sections were cut coronally through the entire septo-temporal axis of the hippocampus. The sections were collected serially in 24-well plates filled with PB. Every 20th section through the entire hippocampus was then selected from six naive control rats and six GWI-rats and processed for PV immunohistochemistry, which visualized the PV+ interneurons in different subfields of the hippocampus. Additional series of sections were processed for NPY, SS (every 20th) and DCX (every 15th) immunohistochemistry for visualization of NPY+ and SS+ interneurons in different regions of the hippocampus, and DCX+ newly born neurons in the SGZ-GCL of the DG (*n* = 6 animals/group).

### PV immunohistochemistry

The procedure employed for PV immunohistochemistry is detailed in our previous report (Shetty and Turner, 1998). Briefly, the sections were first processed for etching, which involved immersion of sections in phosphate buffered saline (PBS) solution containing 20% methanol and 3% hydrogen peroxide for 20 min. Sections were next rinsed three times in PBS, treated with 10% normal horse serum in PBS containing 0.1% Triton-X 100, and incubated overnight in a mouse anti-parvalbumin antibody solution (1:2000 in PBS, Sigma). Subsequently, the sections were bathed thrice in PBS, treated with the biotinylated anti-mouse IgG solution (1:200, Vector) for 60 min, washed three times in PBS, and treated with the avidin-biotin complex (ABC) reagent (Vector) for 60 min, as per manufacturer’s instructions. The peroxidase reaction was developed using diaminobenzidine (DAB) as chromogen (Vector). The sections were placed on gelatin coated slides, dried overnight, dehydrated, cleared, and cover slipped with DPX.

### NPY and SS immunohistochemistry

A detailed methodology employed is described elsewhere (Scharfman et al., 2002; Hattiangady et al., 2005). Succinctly, the sections were first processed for etching through incubation in 0.1 M Tris buffer (TB) containing 1% hydrogen peroxide for 30 min. Following this, the sections were bathed three times in TB, and treated consecutively with TB containing 0.1% Triton X-100 (Tris A; 10 min) and TB containing 0.1% Triton X-100 and 0.005% bovine serum albumin (Tris B; 10 min). Subsequently, the sections were treated with a blocking solution comprising 10% normal goat serum in Tris B for 45 min. Next, the sections were rinsed thoroughly in Tris A and Tris B and treated with a rabbit anti-NPY antibody or a rabbit anti-SS antibody (1:1000, Peninsula laboratories, San Carlos, CA, USA) for 48 h at 4°C. The sections were then washed consecutively in Tris A and Tris B buffers, and treated with biotinylated goat anti-rabbit IgG (1:1000; Vector) for 45 min. The sections were next washed consecutively in Tris A and Tris D (0.5 M TB comprising 0.1% Triton X-100 and 0.005% bovine serum albumin), and treated with the ABC reagent (Vector) solution diluted in Tris D (1:1000) for 60 min. The tissue-bound peroxidase was developed using vector gray (Vector) as chromogen. The sections were air dried following their placement on gelatin coated slides, dehydrated, cleared, and cover slipped with DPX.

### Doublecortin immunohistochemistry

The methodology employed for DCX immunohistochemistry is detailed in our previous reports (Rao and Shetty, 2004; Rao et al., 2005). Briefly, the sections were etched and washed three times in PBS, immersed in 3% normal horse serum in PBS containing 0.1% Triton-X 100 for 30 min, and incubated for 24 h in an affinity purified goat polyclonal anti-DCX antibody solution (1:200 in PBS, Santa Cruz Biotechnology, Santa Cruz, CA, USA). Subsequently, the sections were processed using the ABC method.

### Counting of PV+, NPY+ and SS+ interneurons in the DG and CA1 and CA3 subfields and DCX+ newly born neurons in the SGZ-GCL

In every animal belonging to naive control and GWI groups (*n* = 6/group), numbers of PV+, NPY+ and SS+ interneurons were stereologically counted for the DG and the CA1 and CA3 subfields of the hippocampus. On the other hand, the DCX+ newly born neurons were stereologically counted for the SGZ-GCL region. All counts utilized every 15th or 20th section through the entire septo-temporal axis of the hippocampus and the optical fractionator method available in the StereoInvestigator system (Microbrightfield Inc.). The StereoInvestigator system consisted of a color digital video camera (Optronics Inc.) interfaced with a Nikon E600 microscope.

### Optical fractionator cell counting

A detailed protocol employed for counting using the optical fractionator method is described in our preceding publications (Rao and Shetty, 2004; Hattiangady et al., 2011). Interneurons that are PV+, NPY+ or SS+ in the DG or CA1 and CA3 subfields of the hippocampus and DCX+ newly born neurons in the SGZ-GCL were counted from 50–600 frames selected through a systematic random sampling scheme in every chosen section using a 100× oil immersion lens. All counts in this study used a counting frame that measured 40 × 40 μm. To count interneurons, the outlines of different hippocampal regions (the DG and the CA1 and CA3 subfields) were initially demarcated in every section via tracing function in StereoInvestigator. The numbers and sites of counting frames and the counting depth for each section was next established by entering factors such as the grid size, the thickness of the top guard zone (4 μm) and the optical dissector height (8 μm). A motorized stage controlled through the software then permitted every section to be evaluated at each of the counting frame sites. All PV+, NPY+, SS+ or DCX+ neurons that could be located within the 8-μm section depths in every site were counted. The same methodology was followed for all sections. The software program then calculated the total number of PV+, NPY+ SS+ or DCX+ neurons per each chosen region by utilizing the optical fractionator formula, *N* = 1/ssf.1/asf.1/hsf.EQ-. The acronym ssf signifies the section-sampling fraction, which were 20 for counts of PV+, NPY+ and SS+ interneurons and 15 for counts of DCX+ newly born neurons. The abbreviation asf denotes the area-sampling fraction, which was computed by dividing the area sampled with the total area of the respective subfield (i.e., the sum of subfield areas sampled in every section). The ellipsis hsf stands for the height sampling fraction, which was measured by dividing the height sampled (i.e., 8 μm in this study) with the actual section depth. EQ- denotes the total count of neurons sampled for each subfield. The software program also facilitated the computation of data as density per mm^3^ volume of the tissue sampled.

### Data analyses

The total numbers as well as densities per mm^3^ volume of tissue were calculated for PV+, NPY+ and SS+ interneurons, and DCX+ newly born neurons for the chosen region/s in every animal before calculating means ± standard errors (S.E.M.) for the two groups. The PV+, NPY+, SS+ total interneuron counts and densities for different hippocampal regions and DCX+ newly born neuron numbers and densities in the SGZ-GCL region of age-matched naive control rats (*n* = 6) and GWI-rats (*n* = 6) were then compared through two-tailed, unpaired Student’s *t*-test.

## Results

### GWI-rats displayed greatly reduced numbers of PV+ interneurons in the DG

**Figure 1:**
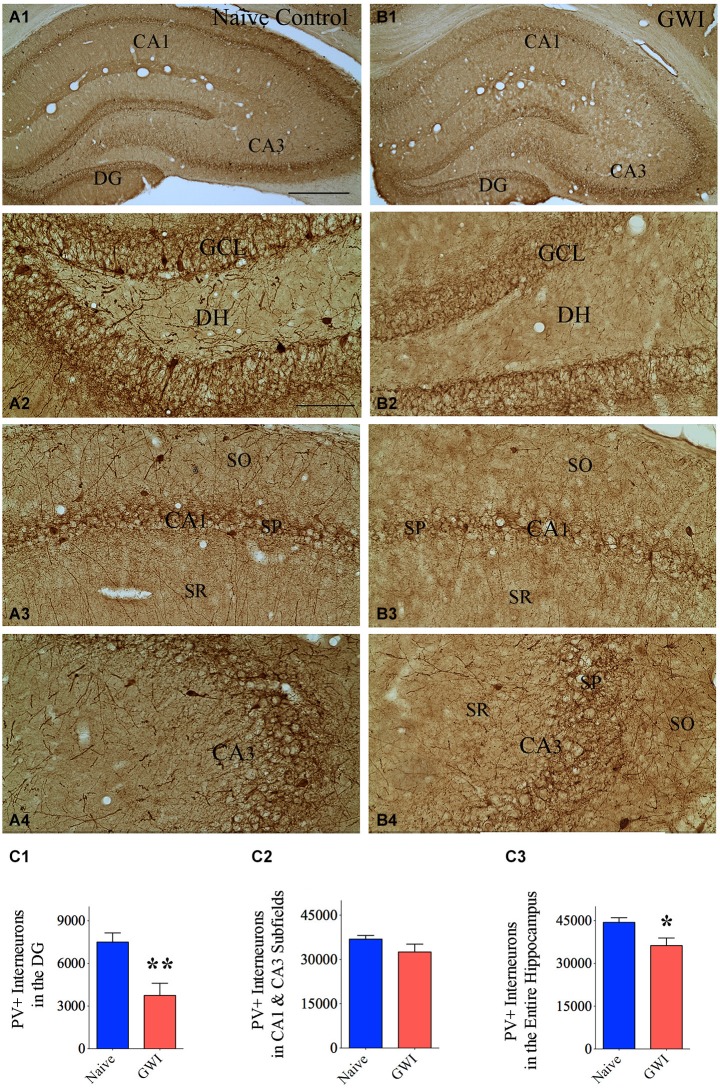
**Animals exposed to Gulf War illness related (GWIR) chemicals and stress exhibit loss of parvalbumin-positive (PV+) interneurons in the hippocampus**. Panels **(A1)** and **(B1)** illustrate the distribution of PV+ interneurons in different subfields of the hippocampus of an age-matched naive control rat **(A1)** and a rat that was exposed to GWIR-chemicals and stress 3 months earlier **(B1)**. Panels **(A2–A4)** show magnified views of the dentate gyrus (DG), the CA1 subfield and the CA3 subfield from the panel **(A1)**. Panels **(B2–B4)** show magnified views of the DG, the CA1 subfield and the CA3 subfield from the panel **(B1)**. Paucity of PV+ interneuron cell bodies and processes are seen in the DG and CA1 regions of a GWI-rat **(B2, B3)**, in comparison to respective regions from a naive control rat **(A2, A3)**. Scale bar **(A1)** and **(B1)** = 500 μm; **(A2–A4)** and **(B2–B4)** = 100 μm. Bar charts in **(C1–C3)** compare numbers of PV+ interneurons in the DG **(C1)**, CA1 and CA3 subfields **(C2)** and the entire hippocampus **(C3)** between naive control rats and GWI-rats. *p < 0.05; **p < 0.01 (two tailed, unpaired Student’s *t*-test). DH, dentate hilus; GCL, granule cell layer; SO, stratum oriens, SP, stratum pyramidale; SR, stratum radiatum.

Immunohistochemical staining of sections with an antibody against PV allowed examination of the distribution of PV-expressing GABA-ergic interneurons in the DG and the CA1 and CA3 subfields of both naive control rats and GWI-rats (Figures 1A1,B1). In the DG of naive control animals, significant numbers of PV+ cell bodies were clearly seen at the junction of the dentate hilus and the dentate GCL (presumably the GABA-ergic basket cells in the DG, Figure 1A2). In contrast, the DG of GWI-rats displayed greatly reduced occurrence of such interneurons. Some sections along the septo-temporal axis of the hippocampus showed even a complete absence of such cells (Figures 1B1,B2). Reduced PV+ interneuron population in the hippocampus of GWI-rats was also evidenced through a reduced density of PV+ fibers (dendrites and axons) in the dentate hilus and strata oriens, pyramidale and radiatum of CA1 and CA3 subfields, in comparison to naive control animals (Figures 1A2–A4,B2–B4). Stereological quantification revealed that GWI-rats displayed 50% reduction in the total number of PV+ interneurons in the DG, in comparison to naive control animals (*p* < 0.01, Figure 1C1). Although the reduction observed in total number for the CA1 and CA3 subfields of GWI-rats was not significant (12% reduction, *p* > 0.05, Figure 1C2), the overall reduction in PV+ interneurons in GWI-rats amounted to 18% when the hippocampus was taken in its entirety (*p* < 0.05, Figure 1C3). Analyses of the density of PV+ interneurons per mm^3^ volume of tissue also revealed 50% reduction in the DG (*p* < 0.05, Table 1). Thus, GWI-rats exhibit a major loss of PV+ interneurons in the DG, evidenced through reductions in the total number as well as the density per unit volume.

**Table 1:** **Density of parvalbumin (PV) positive interneurons in the hippocampus**.

Area of the hippocampus analyzed	Density per mm^3^ (Mean + S.E.M)		*p*-value*
	Naive rats	GWI rats
DG	2125 ± 180	1051 ± 300	*p* < 0.05
CA1 and CA3 subfields	3064 ± 204	2968 ± 220	*p* > 0.05

GWI, Gulf War illness; DG, dentate gyrus; *two-tailed, unpaired Student’s t-test.

### GWI-rats demonstrated diminished numbers of NPY+ interneurons in the CA1 and CA3 subfields of the hippocampus

**Figure 2:**
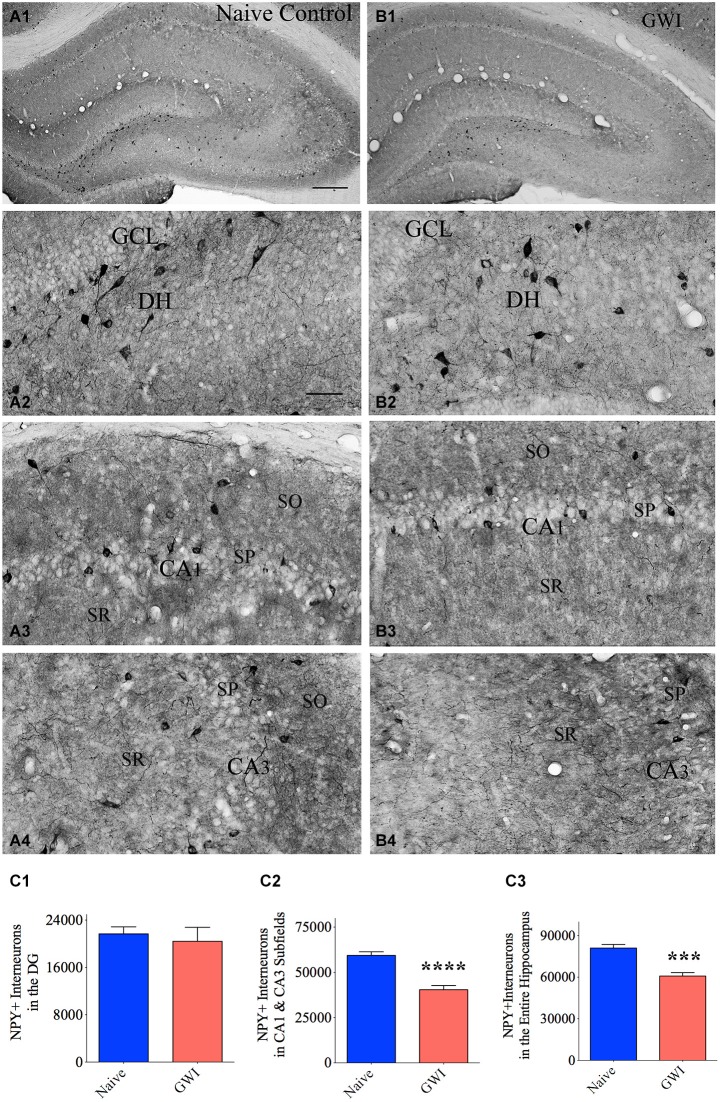
**Exposure to Gulf War illness related (GWIR) chemicals and stress causes loss of the neuropeptide Y-positive (NPY+) interneurons in the hippocampus**. Panels **(A1)** and **(B1)** illustrate the distribution of NPY+ interneurons in different subfields of the hippocampus of an age-matched naive control rat **(A1)** and a rat that was exposed to GWIR-chemicals and stress 3 months earlier **(B1)**. Panels **(A2–A4)** show magnified views of the dentate gyrus (DG), the CA1 subfield and the CA3 subfield from the Panel **(A1)**. Panels **(B2–B4)** show magnified views of the DG, the CA1 subfield and the CA3 subfield from the Panel **(B1)**. Reduced density of NPY+ interneuron cell bodies is apparent in the CA1 and CA3 subfields of a GWI rat **(B3, B4)**, in comparison to respective regions from a naive control rat **(A3, A4)**. Scale bar **(A1)** and **(B1)** = 500 μm; **(A2–A4)** and **(B2–B4)** = 100 μm. Bar charts in **(C1–C3)** compare numbers of NPY+ interneurons in the DG **(C1)**, CA1 and CA3 subfields **(C2)** and the entire hippocampus **(C3)** between naive control rats and GWI-rats. ***p < 0.001; ****p < 0.0001 (two tailed, unpaired Student’s *t*-test). DH, dentate hilus; GCL, granule cell layer; SO, stratum oriens, SP, stratum pyramidale; SR, stratum radiatum.

The composition of NPY+ interneurons in the DG and the CA1 and CA3 subfields of naive control rats and GWI-rats was examined through immunohistochemical staining of sections using an antibody against NPY (Figures 2A1,B1). In the DG of both groups, somata of NPY+ interneurons were conspicuously seen in the dentate hilus (Figures 2A2,B2). However, the CA1 and CA3 subfields of GWI-rats exhibited reduced density of NPY+ interneurons in the strata oriens, pyramidale and radiatum, in comparison to their counterparts in naive control animals (Figures 2A3,A4,B3,B4). Stereological quantification revealed that the total number of NPY+ interneurons in the DG was comparable between naive control rats and GWI-rats, implying that NPY+ interneuron population in the DG is mostly resistant to GWIR chemicals and mild stress exposures (*p* > 0.05, Figure 2C1). However, the total number of NPY+ interneurons in the CA1 and CA3 subfields was reduced in GWI-rats, in comparison to naive control animals (32% reduction, *p* < 0.0001; Figure 2C2). There was also an overall reduction in the total number of NPY+ interneurons when the entire hippocampus was taken in entirety (25% reduction, *p* < 0.001; Figure 2C3). However, the density of NPY+ interneurons per mm^3^ volume of tissue showed only 16% reduction in CA1 and CA3 subfields (*p* > 0.05, Table 2). The discrepancy in percentage reductions between total numbers and the density per unit volume reflects some diminution in the overall volume of CA1 and CA3 subfields in GWI-animals as reported in our previous study (Parihar et al., 2013). Thus, GWI-rats display a greater reduction in the total number and a moderate reduction in the density of NPY+ neurons in CA1 and CA3 subfields of the hippocampus.

**Table 2:** **Density of neuropeptide Y (NPY) positive interneurons in the hippocampus**.

Area of the hippocampus analyzed	Density per mm^3^ (Mean + S.E.M)		*p*-value*
	Naive rats	GWI rats
DG	5283 ± 424	5468 ± 639	*p* > 0.05
CA1 and CA3 subfields	4695 ± 268	3959 ± 341	*p* > 0.05

GWI, Gulf War illness; DG, dentate gyrus; *two-tailed, unpaired Student’s t-test.

### GWI-rats exhibited no loss of SS+ interneurons in the hippocampus

**Figure 3:**
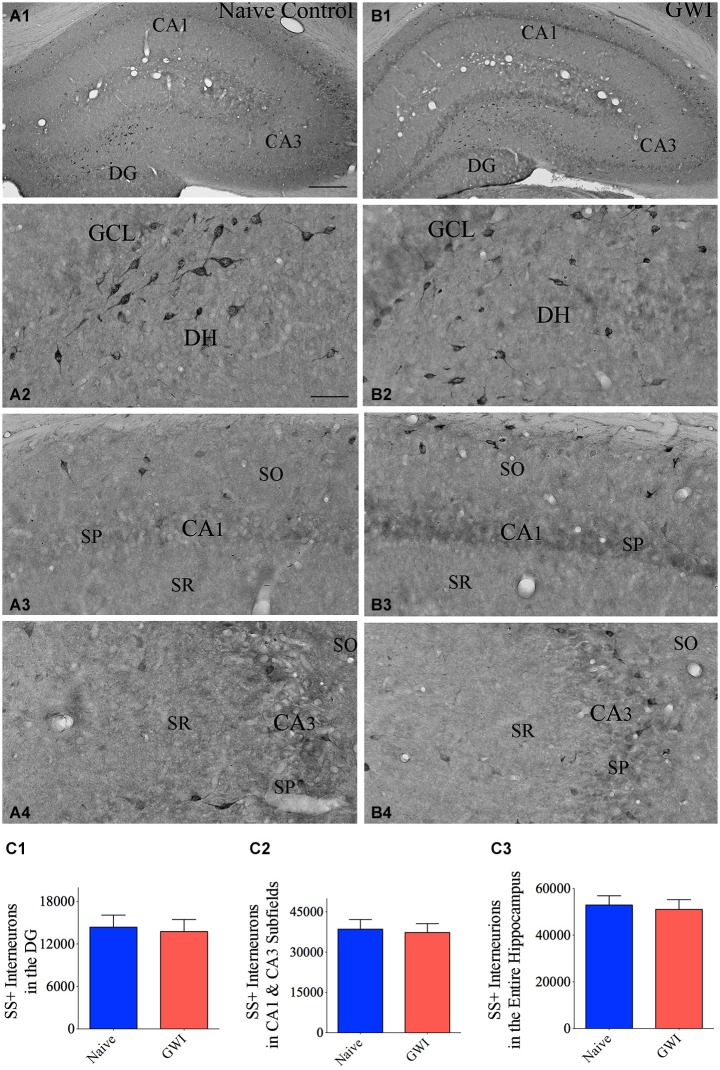
**Exposure to Gulf War illness related (GWIR) chemicals and stress does not reduce somatostatin-positive (SS+) interneurons in the hippocampus**. Panels **(A1)** and **(B1)** illustrate the distribution of SS+ interneurons in various regions of the hippocampus of an age-matched naive control rat **(A1)** and a rat that was exposed to GWIR-chemicals and stress 3 months earlier **(B1)**. Panels **(A2–A4)** show magnified views of the dentate gyrus (DG), the CA1 subfield and the CA3 subfield from the panel **(A1)**. Panels **(B2–B4)** show magnified views of the DG, the CA1 subfield and the CA3 subfield from panel **(B1)**. Note that densities of SS+ interneuron cell bodies in all hippocampal regions appear comparable between a Gulf War illness (GWI)-rat **(B2–B4)** and a naive control rat **(A2–A4)**. Scale bar **(A1)** and **(B1)** = 500 μm; **(A2–A4)** and **(B2–B4)** = 100 μm. Bar charts in **(C1–C3)** compare numbers of SS+ interneurons in the DG **(C1)**, CA1 and CA3 subfields **(C2)** and the entire hippocampus **(C3)** between naive control rats and GWI-rats. DH, dentate hilus; GCL, granule cell layer; SO, stratum oriens, SP, stratum pyramidale; SR, stratum radiatum.

Immunohistochemical staining of sections using an antibody against SS showed the distribution of cell bodies of SS+ interneurons in the DG, the stratum oriens of CA1 subfield, and the stratum pyramidale of CA3 subfield in both naive control rats and GWI-rats (Figures 3A1–B4). Densities of SS+ interneurons appeared similar between the two groups of rats for the DG as well as CA1 and CA3 subfields (Figures 3A1–B4). However, the soma size of SS+ positive neurons in the dentate hilus appeared smaller in GWI-rats, in comparison to naive control rats (Figures 3B2,C2). Stereological quantification revealed no differences in numbers of SS+ interneurons between naive control rats and GWI-rats for the DG, CA1 and CA3 subfields and the entire hippocampus (Figures 3C1–C3). Analyses of the density of SS+ interneurons per mm^3^ volume of tissue also showed a similar trend (Table 3). Thus, GWI-rats display no reductions in total number or density of SS+ interneurons in the hippocampus.

**Table 3:** **Density of somatostatin (SS) positive interneurons in the hippocampus**.

Area of the hippocampus analyzed	Density per mm^3^ (Mean + S.E.M)		*p*-value*
	Naive rats	GWI rats
DG	3696 ± 361	4015 ± 583	*p* > 0.05
CA1 and CA3 subfields	2864 ± 242	3160 ± 217	*p* > 0.05

GWI, Gulf War illness; DG, dentate gyrus; *two-tailed, unpaired Student’s t-test.

### GWI-rats displayed decreased neurogenesis in the hippocampus

**Figure 4:**
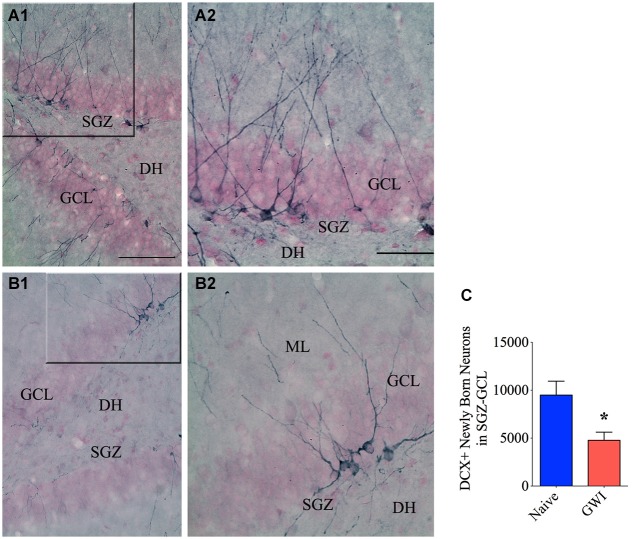
**Exposure to Gulf war illness related (GWIR) chemicals and stress leads to diminished neurogenesis in the hippocampus**. Panels **(A1)** and **(B1)** illustrate the distribution of doublecortin-positive (DCX+) interneurons in the subgranular zone-granule cell layer (SGZ-GCL) of the DG from an age-matched naive control rat **(A1)** and a rat that was exposed to Gulf War illness related (GWIR) chemicals and stress 3 months earlier **(B1)**. The panels **(A2)** and **(B2)** are magnified views of marked regions from **(A1)** and **(B1)** showing the morphology of DCX+ newly born neurons. Scale bar **(A1)** and **(B1)** = 100 μm; **(A2)** and **(B2)** = 50 μm. DH, dentate hilus; ML, molecular layer. The bar chart **(C)** compares numbers of newly born (DCX+) neurons in the DG between naive control rats and rats exposed to GWIR-chemicals and stress. *p < 0.05.

Because of the suggested links between the integrity of PV+ and NPY+ interneurons and neurogenesis in the DG, we investigated the status of neurogenesis from both naive control and GWI-rats via stereological counting of DCX+ newly born neurons in the SGZ-GCL. Immunostaining for DCX visualized newly born neurons in both age-matched naive control rats and GWI-rats (Figures 4A1–B2). Stereological quantification revealed the occurrence of ~9492 newly born neurons (Mean ± S.E.M = 9492 ± 1451, *n* = 6) per hippocampus in naive control animals. However, GWI-rats demonstrated a clearly reduced number of newly born neurons per hippocampus (4775 ± 851, *n* = 6, *p* < 0.05, 50% reduction, Figure 4C). Analyses of the density per 0.1 mm^3^ volume of SGZ-GCL tissue also revealed a similar trend (Naive rats, 1383 ± 155; GWI-rats, 708 ± 169; *p* < 0.05, 51% reduction). Thus, a clear association was found between the decreased neurogenesis and PV+ interneuron loss in the DG of GWI-rats.

## Discussion

This study provides new evidence that combined exposure to low doses of GWIR chemicals and mild stress for 4 weeks reduces the overall number as well as the density of PV-expressing GABA-ergic interneurons in the DG and NPY-expressing interneurons in the CA1 and CA3 subfields of the hippocampus. This study also demonstrates that PV and NPY interneuron deficiency in the hippocampus co-exists with greatly decreased neurogenesis in GWI-rats. The mechanism by which PV+ and NPY+ interneuron numbers decline after exposure to GWIR chemicals and stress remains to be determined however. Persistent inhibition of AChE in the exposure and early post-exposure periods may be involved because the GWIR chemicals are AChE inhibitors and persistent exposure to even low doses of AChE inhibitors can alter the regulation of cholinergic function and increase oxidative stress (Golomb, 2008). Although the current study did not measure AChE activity, a previous study using the same prototype of GWI has showed a significant reduction in AChE activity in the brain following exposure to GWIR chemicals and mild stress (Abdel-Rahman et al., 2002). Additionally, increased oxidative stress and low-level inflammation in the hippocampus observed after exposure to GWIR chemicals and stress (Parihar et al., 2013; Shetty et al., 2014) may be also contributing to interneuron loss. Additional longitudinal studies will be needed in the future to comprehend these issues fully. Nonetheless, the implications of this study go further beyond the GWI issue as exposure to insecticides or pesticides alone may mimic the effects observed in this study and lead to cognitive decline (Cañadas et al., 2005; Suarez-Lopez et al., 2013).

### Consequences of reduced PV+ interneuron numbers in the hippocampus of GWI-rats

In the hippocampus, GABA-ergic interneurons expressing the calcium binding protein PV are mainly observed in the dentate GCL, the dentate hilus, and strata oriens and pyramidale of CA1–CA3 subfields (Celio and Heizmann, 1981; Kosaka et al., 1987). These interneurons play important roles in maintaining hippocampus function. Hence, greatly reduced number and density of PV+ interneurons in the DG of GWI-rats has multiple functional implications. The DG is believed to be a gateway to the hippocampus, as the connectivity between DG granule cell dendrites and perforant path axons from the entorhinal cortex makes the first leg of the hippocampus tri-synpatic pathway (Amaral et al., 2007). The activity of excitatory granule cells is regulated by GABA-ergic interneurons in the DG. Although there are numerous subpopulations of GABA-ergic interneurons in the DG, fast-spiking PV-expressing interneurons located at the junction of the dentate hilus and the GCL (i.e., basket cells) and the dentate hilus play a major role in perisomatic inhibition of granule cells, as these interneurons are capable of providing both feed-back and feed-forward inhibition to dentate granule cells (Struble et al., 1978; Freund and Buzsáki, 1996). While the feedback inhibition is mediated through their innervation by recurrent glutamatergic mossy fibers of granule cells (Blasco-Ibáñez et al., 2000), the feed-forward inhibition is mediated via innervation of their distal dendrites by perforant path axons from the entorhinal cortex (Zipp et al., 1989; Nieto-Gonzalez and Jensen, 2013). Furthermore, feedback microcircuits involving PV+ interneurons have several functions beyond simple inhibition (Hu et al., 2014). It has been proposed that feedback inhibition facilitates the firing of dentate granule cells with the strongest input and inhibition of remaining dentate granule cells (de Almeida et al., 2009a,b). This computation is considered to have particular importance in the DG as this may promote selective activation of only certain numbers of neurons (Pernía-Andrade and Jonas, 2014) and pattern separation function (Leutgeb et al., 2007). Thus, PV+ interneurons contribute to advanced computations in microcircuits and neuronal networks (Hu et al., 2014).

Reduced numbers of PV+ interneurons in the DG affects network properties, which can lead to hippocampus hyperexcitability (Schwaller et al., 2004; Andrioli et al., 2007). Indeed, studies in epilepsy models have shown that loss of PV+ interneurons in the DG greatly reduces the inhibition of dentate granule cells and contributes to epileptogenesis (Kobayashi and Buckmaster, 2003; Sloviter et al., 2003; Ben-Ari, 2006). From this perspective, a substantial loss of PV+ interneurons observed in the DG of GWI-rats may induce hippocampus hyperexcitability. Furthermore, while GWI-rats did not show reduced numbers or densities of PV+ interneurons in CA1 and CA3 subfields, qualitative observations revealed reduced density of PV+ axons in the CA1 subfield of GWI-rats, implying some impairment among surviving PV+ interneurons. As selective removal of PV+ interneurons in the CA1 subfield induces spatial working memory dysfunction, loss of PV+ axons observed in the CA1 subfield can cause working memory impairment (Murray et al., 2011). Likewise, the activity of PV+ interneurons is vital for synchronizing the hippocampal pyramidal neurons during network oscillations (Klausberger et al., 2005). Because hippocampal network oscillations systematize the activity of large neuronal populations at diverse time scales and offer conditions for adaptive management of networks during information encoding, processing and storage, the PV+ interneurons have functional relevance for contributing to cognitive processes handled by the hippocampus (Korotkova et al., 2010). Considering these and because hyperexcitability is known to interfere with cognitive function (Spiegel et al., 2013), it is plausible that the loss of PV+ interneurons in the hippocampus is one of several factors underlying cognitive impairments observed in rat models of GWI and in veterans afflicted with GWI (Odegard et al., 2013; Parihar et al., 2013; Hattiangady et al., 2014).

Decreased numbers of PV+ interneurons in the DG can also influence the extent of neurogenesis. Neurogenesis in the DG involves several steps. Nestin+ primary neural stem cells (NSCs, also known as radial glia-like cells) occasionally divide to generate a highly proliferative intermediate progenitor cell population (transient amplifying cells), which give rise to large numbers of DCX+ immature neurons. Ambient GABA in the microenvironment tonically activates these immature neurons, which is then followed successively by depolarizing GABA-ergic and glutamatergic synaptic inputs (Song et al., 2014). Recent studies have demonstrated that PV+ interneurons in the DG suppress the activation of quiescent NSCs by releasing non-synaptic GABA under normal conditions (Song et al., 2012). Such regulation mediated through γ2-containing GABA_A_Rs expressed on NSCs is considered important, as it prevents the depletion of primary NSCs through rapid division (Song et al., 2013). Furthermore, specific activation of PV+ interneurons has been found to promote the survival of proliferating DCX+ immature neurons through establishment of immature synapses between axons of PV+ interneurons and newly born neurons. These results imply that GABA signaling mediated by the activity of PV+ interneurons is critical for controlling several important phases of hippocampal neurogenesis: primary NSC quiescence to prevent their loss through excessive division and the survival and maturation of immature neurons to maintain the ability to insert new neurons into the hippocampal circuitry for making new memories (Song et al., 2014). From these viewpoints, it is plausible that PV+ interneuron deficiency observed in the DG of GWI-rats can considerably impair neurogenesis. Indeed, our quantification revealed ~50% reduction in neurogenesis in GWI-rats that displayed PV+ interneuron loss. Thus, reduced numbers of PV+ interneurons in GWI-rats may be having an additive influence to other detrimental effects in the microenvironment such as an increased oxidative stress and chronic low-level inflammation (Parihar et al., 2013; Shetty et al., 2014). Thus, considerably diminished PV+ interneuron subpopulation in the DG may be one of the major neuropathological hallmarks of GWI, as this can contribute to reduced neurogenesis, impaired cognitive function and hippocampus hyperexcitability.

### Implications of reduced NPY+ interneuron numbers in the hippocampus of GWI-rats

Our analyses showed that the overall number and density of NPY+ interneuron population remained stable in the DG but decreased in the CA1 and CA3 subfields of GWI-rats. This may have several consequences because NPY+ interneurons in the hippocampus play an important role in regulating the activity of hippocampal circuitry. The NPY released from these interneurons modulates the activity of excitatory neurons by consistently hyperpolarizing and reducing their spike frequency (Fu and van den Pol, 2007) and by inhibiting the glutamate release on to principal hippocampal neurons (Colmers and Bleakman, 1994). The NPY is also an endogenous anti-seizure compound, which has been evidenced through its upregulation in response to status epilepticus, occurences of seizure activity with reduced NPY levels and reduced epileptiform-like activity with NPY treatment in epilepsy prototypes (Baraban et al., 1997; Klapstein and Colmers, 1997; Patrylo et al., 1999; Vezzani et al., 1999; Sperk et al., 2007). Furthermore, the NPY has a role in hippocampal neurogenesis and functions such as learning, memory and mood (Zaben and Gray, 2013). Hence, reduced NPY levels in the hippocampus of GWI-rats may considerably interfere with the hippocampus function. Particularly, this alteration can contribute to hippocampus hyperexcitability and impaired learning and memory function. While the hippocampus hyperexcitability in GWI is currently unknown, increased oxidative stress and chronic low-level inflammation in the hippocampus and persistent cognitive dysfunction have been observed in animal models of GWI and/or veterans afflicted with GWI (Odegard et al., 2013; Parihar et al., 2013; Hattiangady et al., 2014; Shetty et al., 2014). From these perspectives, drugs capable of dampening hyperactivity via improved inhibitory neurotransmission may prove to be beneficial, as such approach has been found to be useful for alleviating age-related memory impairments associated with hippocampus hyperexcitability and GABA-ergic interneuron dysfunction (Koh et al., 2010, 2013).

## Conclusions

Our findings establish for the first time that PV+ interneurons in the DG and NPY+ interneurons in CA1 and CA3 subfields of the hippocampus decline in total number as well as density per unit volume of the tissue in a model of GWI. The results also uncover that decreased interneuron numbers are coupled with greatly decreased neurogenesis in the hippocampus of GWI-rats. Because PV+ and NPY+ interneurons play roles in maintaining normal cognitive function, regulating the extent of neurogenesis, and controlling the activity of excitatory neurons, their deficiency is likely among the major factors contributing to impaired cognitive function seen in GWI.

## Author contributions

Tarick Megahed contributed to stereological counts of interneurons, data analyses, the preparation of figures and figure composites, and prepared an initial draft of the manuscript text. Bharathi Hattiangady gave input to the experimental design, and immunohistochemistry, performed some stereological counts and data analyses, prepared figure composites and contributed to manuscript writing. Bing Shuai performed chemical and stress exposures to animals, tissue processing and immunohistochemistry. Ashok K. Shetty conceptualized the experimental design, analyzed and interpreted data, revised figure composites and wrote the final version of the manuscript text. All authors gave input to the manuscript text and approved the final version of the manuscript.

## Conflict of interest statement

The authors declare that the research was conducted in the absence of any commercial or financial relationships that could be construed as a potential conflict of interest.
